# Staging the clinically negative neck in T1-T2 oral cancers: USgFNAC and sentinel node biopsy

**DOI:** 10.1186/1470-7330-14-S1-S10

**Published:** 2014-10-09

**Authors:** S Arya, S Datta, P Chaturvedi

**Affiliations:** 1 Tata Memorial Centre, Mumbai, India

## Background

A meta-analysis by deBondt declared ultrasound guided fine needle aspiration cytology (USgFNAC) as the method with the highest diagnostic odds ratio for detecting metastatic nodes in head and neck squamous carcinoma (HNSCC). Sentinel node biopsy (SNB) is another emerging method for evaluating neck nodes in HNSCC with reported high sensitivity and negative predictive value (NPV).

## Objective

The aim of this study was to compare USgFNAC with SNB for the preoperative evaluation of the clinically negative neck in T1 & T2 oral cavity squamous cell carcinoma (OCSCC).

## Methodology

This is a prospective observational study in 51 patients with T1-T2 N0 OCSCC. Pre-operative ultrasonography (US) of the neck was performed in all patients. USgFNAC was performed in patients where US was reported as indeterminate or positive. SNB was performed in all patients and was followed by elective neck dissection (END). The sensitivity, specificity, positive predictive value (PPV) and NPV of SNB, USgFNAC and US were calculated considering END and histopathology (HP) as the gold standard.

## Results

The incidence of occult metastasis on HP was 26.4%. The sensitivity, specificity, PPV and NPV were 71.4%, 100%, 100% and 90.2% for SNB; 50%, 82%, 50% and 82% for US; and 14.3%, 100%, 100% and 76.5% for USgFNAC respectively.

## Conclusions and relevance

The meta-analysis by deBondt with reported highest accuracy for USgFNAC included both clinically positive and negative necks. This study evaluated USgFNAC and SNB in the clinically negative neck in T1-T2 oral cancers and found SNB clearly superior to USgFNAC.

